# Antimicrobial activity, toxicity and selectivity index of two biflavonoids and a flavone isolated from *Podocarpus henkelii* (Podocarpaceae) leaves

**DOI:** 10.1186/1472-6882-14-383

**Published:** 2014-10-08

**Authors:** Victor P Bagla, Lyndy J McGaw, Esam E Elgorashi, Jacobus N Eloff

**Affiliations:** Phytomedicine Programme, Department of Paraclinical Sciences, Faculty of Veterinary Science, University of Pretoria, Private Bag X04, Onderstepoort, 0110 South Africa

**Keywords:** Selectivity index, Biflavonoids, Antimicrobial activity, Cytotoxicity, Mutagenicity

## Abstract

**Background:**

Different parts of *Podocarpus henkelii* have been used in many cultures around the world to treat ailments such as cholera, stomach diseases, rheumatism, cancer, canine distemper in dogs and gall sickness in cattle. The aim of this study was to evaluate the biological activity and toxicity of isolated compounds from *Podocarpus henkelii* after an earlier study indicated a promising activity in crude extracts against viral pathogens of veterinary importance.

**Methods:**

The antibacterial and antifungal activity of two biflavonoids 7, 4’, 7”, 4”’-tetramethoxy amentoflavone (TMA), isoginkgetin (IGG) and podocarpus flavone–A (PFA) isolated from the leaves of *Podocarpus henkelii* were determined using a serial microplate dilution method with tetrazolium violet as growth indicator. The cytotoxicity of compounds TMA and IGG were determined on different cell types using a tetrazolium-based colorimetric cellular assay (MTT). The Ames test was used to determine their mutagenic activities.

**Results:**

TMA had reasonable antifungal activity against *Aspergillus fumigatus* (MIC = 30 μg/ml). IGG had a wide spectrum of activity against four bacterial and two fungal pathogens with much higher selectivity index values obtained for *A. fumigatus* and *Cryptococcus neoformans* (SI > 30). PFA had a broad spectrum of activity against *Enterococcus faecalis* and *Pseudomonas aeruginosa* (SI > 15) and less activity against the two fungal pathogens. In both the cytotoxicity assays and Ames mutagenicity test using *Salmonella typhimurium* strains TA98 and TA100, TMA and IGG had no deleterious effect on the different cell types and did not induce mutations in the Ames test.

**Conclusion:**

Although the antimicrobial activities of the isolated compounds were not that exciting, the compounds had no cytotoxic activity at the highest concentration (1000 μg/ml) tested against all three cell lines. IGG was the most active against *E. coli*, *S. aureus*, *A. fumigatus* and *C. neoformans*, exhibiting both antibacterial and antifungal activity with good selectivity index values. PFA had a broad spectrum of activity against *E. faecalis* and *P. aeruginosa.* The two compounds isolated had low toxicity and no genotoxic activity in the Ames test.

## Background

In nature, different types of plants produce certain chemicals that are naturally toxic to microorganisms. These chemicals produced by plants play an essential role in the natural defence and well-being of plants, and belong to a wide range of classes which include the flavonoids and isoflavonoids [1]. Flavonoids can be classified into flavanones, flavones, flavonols, and biflavones [2]. Biflavonoids are linkages of flavone–flavone, flavanone–flavones or flavanone–flavanone subunits. Naturally occurring flavonoids are polyphenolic compounds which can be found in different parts of plants such as flowers, fruits, nuts, seeds, stems and vegetables. They can also be found in wine, honey and commonly consumed beverages such as tea [3, 4].

Apart from the phytonutritional role of flavonoids in providing beneficial health effects by the alteration of various metabolic processes, these classes of compounds have been acclaimed for their neuroprotective effect [5], antiparasitic activity [6], protective effect against DNA damage and lipoperoxidation [7], antiviral activity [8, 9], antimicrobial activity [10–13], anti-inflammatory activity [14], antioxidant activity [15] and many more.

The biflavonoids podocarpus flavone-A (PFA) and isoginkgetin (IGG) have previously been isolated from *Podocarpus neriifolius* [16]. 7’, 4’, 7”, 4”’-tetramethoxy amentoflavone (TMA) on the other hand has been isolated from *Dacrydium cupressinum* and *Araucaria cooki* [17]. Isoginkgetin has been reported to be less toxic to rat skeletal muscle myoblasts *in vitro* [18] in addition to its inhibition of tumour cell invasion by regulating phosphatidylinositol 3-kinase/Akt -dependent matrix metalloproteinase-9 expression [19]. It also has an inhibitory effect on pre-mRNA splicing [20] and some neuroprotective effects *in vitro* [5].

The presence of diverse molecules represented in the class of biflavonoids and their symmetrical or asymmetrical nature, offers an opportunity for manipulation by synthetic chemists to further potentiate the biological activity of these useful classes of compounds. Despite the promise and potential therapeutic relevance of this class of compounds, very few biflavonoids have been investigated either for their biological activity, toxicity or as leads for the development of new drugs.

To date no information on antimicrobial activity of these biflavonoids is apparently available. Hence, this study was aimed at evaluating the antibacterial and antifungal activity of the compounds, namely isoginkgetin, podocarpus flavone-A and 7’, 4’, 7”, 4”’ -tetramethoxy amentoflavone isolated from *Podocarpus henkelii*. Their cytotoxicity on Vero, bovine dermis and CRFK cells was also assessed in order to determine their selective inhibitory activity, as well as their ability to cause genetic damage by genetic mutations as measured by the Ames test.

## Methods

### Plant extraction, fractionation and column chromatography

Leaves of *Podocarpus henkelii* were collected from the Lowveld National Botanical Garden (NBG) in Nelspruit, Mpumalanga province, South Africa. The tree was identified from the plant label and the identity was confirmed by Mr Willem Froneman. A voucher specimen (PBG818945) was deposited at the Pretoria National Herbarium. The leaf material was air-dried at room temperature and milled into a fine powder. Ground material (500 g) was extracted with acetone (1 g/10 ml) for 24 hours. The supernatant was filtered through Whatman No 1 filter paper. The dried acetone extract (43 g) was subjected to solvent-solvent fractionation into n-butanol, hexane, ethyl acetate, carbon tetrachloride, chloroform and methanol-water fractions.

Following bioassay-guided fractionation, the ethyl acetate, carbon tetrachloride and chloroform fractions containing the highest numbers of antibacterial and antifungal compounds were combined and dried under a stream of air. The mixture was fractionated on a silica gel column (60 × 5 cm) by eluting with a gradient of chloroform: methanol (9:1) to separate the bioactive compounds. Fractions (109, of 30 ml volume) were collected and combined to produce four fractions based on similar compounds present in TLC fingerprints. Compound IGG crystallized out of one fraction and TMA and PFA were obtained on final silica gel chromatography using hexane: ethyl acetate (1:1) as eluant. NMR spectroscopy confirmed the identity of the isolated compounds.

### Determination of minimum inhibitory concentration (MIC) of isolated compounds against bacterial pathogens

The serial microtitre dilution method described by Eloff [21] was used to determine the minimum inhibitory concentration (MIC) of the isolated compounds. The method involves the reduction of *p*-iodonitrotetrazolium violet (INT) (Sigma) to a red formazan by biologically active organisms. Growth inhibition is evident in wells where there is inhibition of reduction of INT to red formazan coloration. Two Gram-positive and two Gram-negative bacterial pathogens were used to determine the activity of the isolated compounds. The bacterial cultures were incubated in Müller-Hinton (MH) broth overnight at 37°C and diluted 1:100 in MH broth before use in the assay. The densities of the bacterial cultures before antimicrobial testing were approximately: 1.5 × 10^10^ cfu/ml *(Enterococcus faecalis),* 2.6 × 10^12^ cfu/ml *(Staphylococcus aureus),* 5.2 × 10^13^ cfu/ml *(Pseudomonas aeruginosa)* and, 3.0 × 10^11^ cfu/ml *(Escherichia coli)*. Two-fold serial dilutions of isolated compounds (1 mg/ml) dissolved in 100% DMSO were prepared in 96-well microtitre plates, and 100 μl of bacterial culture were added to each well. Plates were incubated at 37°C for 10 h in a 100% humidified incubator. After incubation for 10 h, 40 μl of 0.2 mg/ml INT were added and the plates were further incubated for 2 h. MIC readings were recorded after 12 and 24 hours incubation. Solvent controls and 0.1 mg/ml of the standard antibiotic gentamicin (50 mg/ml, Virbac) were included in each experiment.

### Determination of minimum inhibitory concentration (MIC) of isolated compounds against fungal pathogens

In the antifungal bioassay, the method described by Eloff [21] and modified by Masoko et al. [22] using Sabouraud Dextrose (SD) broth as nutrient medium was used to determine the activity of the isolated compounds. Two-fold serial dilutions in sterile distilled water of isolated compounds (initial concentration 1 mg/ml, dissolved in DMSO) were prepared in 96-well microtitre plates. The fungal pathogens used in this study were *Candida albicans*, *Cryptococcus neoformans* and *Aspergillus fumigatus.* The pathogens were obtained from clinical cases of disease in animals prior to treatment, and were kindly provided by the Department of Veterinary Tropical Diseases, Faculty of Veterinary Science, University of Pretoria. *C. albicans* was isolated from a Gouldian finch, *C. neoformans* from a cheetah, and *A. fumigatus* from a chicken. Fungal cultures were transferred from SD agar plates using a sterile swab into fresh SD broth, and 100 μl of this suspension was added to each well. Microtitre plates were incubated at 35°C for 24 to 48 hours. INT was used as an indicator of growth as previously described [22]. Amphotericin B (0.08 mg/ml), a standard antifungal agent, was included as a positive control. Solvent controls were also included in all experiments and each experiment was repeated three times.

### Cytotoxicity assay using MTT

The cytotoxicity of compounds was tested against the Vero monkey kidney cell line, CRFK cells and bovine dermis cells. Cells used in this study were kindly provided by the Department of Veterinary Tropical Diseases, Faculty of Veterinary Science, University of Pretoria. Cells were maintained in minimal essential medium (MEM, Highveld Biological, South Africa) supplemented with 0.1% gentamicin (Virbac) and 5% foetal bovine serum (Adcock-Ingram). Cultures for the assay were prepared from confluent monolayer cells and seeded at a density of 48 × 10^3^ per well in a 96 well microtitre plate and incubated overnight at 37°C in a 5% CO_2_ atmosphere. TMA and IGG (2 mg) were dissolved in 0.1 ml DMSO to produce a stock concentration of 20 mg/ml solution. PFA was not isolated in sufficient quantity to allow cytotoxicity determination. The stock solutions were serially diluted to final concentrations of 200, 150, 100, 50, 20 and 10 μg/ml in growth medium. The medium on sub-confluent monolayer cells grown overnight was removed and cells were exposed to 200 μl of the different concentrations of test compounds in quadruplicate and incubated at 37°C for 5 days. Cell viability was determined using the standard tetrazolium-based colorimetric assay [23]. Before the addition of MTT (3-[4,5-dimethylthiazol-2-yl]-2,5-diphenyltetrazolium bromide), medium on the cells was replaced with fresh culture medium to exclude reduction of the tetrazolium compound by test compounds. Thereafter, 30 μl of 5 mg/ml MTT dissolved in PBS was added to cells and incubated for four hours. The assay is based on mitochondrial dehydrogenase activity, which is assessed by the reductive cleavage of the tetrazolium salt MTT to yield a purple formazan dye due to the succinic dehydrogenase enzyme activity present in living cells. Thereafter, medium was removed and cells washed with PBS. DMSO (50 μl) was added to each well and the absorbance was measured using a Versamax microplate reader at 570 nm. Berberine chloride (Sigma) was used as a positive control, wells containing only cells were the negative control and a solvent control was included. The percentage cell viability following the addition of varying concentrations of the extracts in relation to untreated controls was calculated. The CC_50_ (cytotoxicity) values were calculated as the concentration of compounds resulting in 50% reduction of absorbance compared to untreated cells. Tests were carried out in quadruplicate and each experiment was repeated three times. For the purpose of calculating selectivity index (SI), CC_50_ values greater than 1000 were taken as being 1000. Selective activities of the compounds were calculated as follows:


### Genotoxicity testing of isolated compounds

TMA and IGG were investigated for their potential mutagenic effect using a plate incorporation procedure [24]. The assay was performed using *Salmonella typhimurium* strains TA98 and TA100. Briefly, 100 μl of bacterial stock were incubated in 20 ml of Oxoid Nutrient broth for 16 h at 37°C on an orbital shaker. The overnight culture (0.1 ml) was added to 2 ml top agar (containing traces of biotin and histidine) together with 0.1 ml test solution (pure compounds, solvent control or positive control) and 0.5 ml phosphate buffer (for exposure without metabolic activation). The top agar mixture was poured over the surface of the agar plate and incubated for 48 h at 37°C. Following incubation, the number of revertant colonies (mutants) was counted. All cultures were prepared in triplicate (except the solvent control where five replicates were made) for each assay. The assays were repeated twice. The positive control used was 4-nitroquinoline-1-oxide (4-NQO) at a concentration of 2 μg/ml.

## Results and discussion

### Antibacterial activity of compounds

There were no changes in MIC values with an extended time of incubation, suggesting that the activity was bactericidal rather than bacteriostatic (Tables 1 and 2). The Gram-positive organisms were more sensitive to test compounds than their Gram-negative counterparts. This finding is consistent with reports ascribing the effectiveness of antimicrobial agents against Gram-positive bacteria to the porous nature of the outer peptide-glycan layer [25, 26]. However, with flavonoids, two factors have been reported to be important in their antibacterial activity, namely the lipophilicity of the compounds and the presence of a hydroxyl substitution on the phenolic ring, especially at the 4^th^ and the 5^th^ positions. The lipophilic nature, which is enhanced by increasing the number of methoxy substitutions, is responsible for the trapping of flavonoids in the lipophilic cell wall of the bacteria (mainly Gram-negative). This possibly explains why TMA was the least active of the isolated biflavonoids against all the tested pathogens with MIC range of 130-250 μg/ml. The MIC values of IGG and PFA ranged between 60 and 250 μg/ml. IGG was the most active against all the test pathogens with good activity against *S. aureus* and *E. faecalis* (MIC = 60 μg/ml) and a higher selectivity index value (Table 2). The three compounds have a 5^th^ hydroxy substituent, which possibly explains some measure of activity. PFA, which had the highest number of hydroxy substituents, had a broader spectrum of activity than the other compounds against *E. faecalis* and *P. aeruginosa* (MIC = 60 μg/ml). Previous reports [10] showed that some biflavones with hydroxyl substituents are completely inactive against *M. tuberculosis*. In this study, the high number of hydroxyl substitution of PFA may be responsible for the uptake of this compound by the organisms. This finding is in agreement with previous reports ascribing structural activity relationships of flavonoids with antibacterial activity [27]. However, these factors do not completely explain the activity of biflavonoids because Lin et al. [10] found that the methylation or acetylation of these compounds caused no significant change in their activity in that study. It may therefore be possible that the broad spectrum of activity observed with PFA may be associated with multiple effects rather than with a specific cellular target.

**Table 1:** **Minimum inhibitory concentration values (μg/ml) of isolated compounds against two Gram-positive and two Gram-negative bacteria**

Organism	TMA	IGG	PFA	Gentamicin
*S. aureus*	130	60	130	3
*E. faecalis*	250	60	60	
*E. coli*	250	130	250	6
*P. aeruginosa*	250	130	60	

(There were no differences after 12 and 24 h incubation).

**Table 2:** **Selectivity index values of compounds against bacterial pathogens**

	TMA	IGG	PFA
*S. aureus*	7.69	16.67	7.69
*E. faecalis*	4.00	16.67	16.67
*E. coli*	4.00	7.69	4.00
*P. aeruginosa*	4.00	7.69	16.67

(There were no differences after 12 and 24 h incubation).

### Antifungal activity of compounds

The activities of the test compounds against fungal pathogens are represented in Tables 3 and 4. The trend of activity did not follow the pattern observed in the antibacterial studies. The best antifungal activity was obtained with IGG against *A. fumigatus* and *C. neoformans* with MIC of 30 μg/ml and a selectivity index value great than 30. A similar result was obtained for TMA against *A. fumigatus*. PFA was less active against the test fungal pathogens with MIC ranging between 130 and 250 μg/ml.

**Table 3:** **Minimum inhibitory concentration values (μg/ml) of compounds against selected fungal pathogens after 24 and 48 h incubation**

Organism	Time (h)	TMA	IGG	PFA	Amp-B
*C. albicans*	24	130	250	250	40
*A. fumigatus*		30	30	250	80
*C. neoformans*		130	30	130	20
*C. albicans*	48	250	250	250	
*A. fumigatus*		30	30	250	
*C. neoformans*		130	30	130	

Amp-B = Amphotericin B.

**Table 4:** **Selectivity index values of compounds against fungal pathogens after 24 and 48 h incubation**

	Time (h)	TMA	IGG	PFA
*C. albicans*	24	7.69	4.00	4.00
*A. fumigatus*		33.33	33.33	4.00
*C. neoformans*		7.69	33.33	7.69
*C. albicans*	48	4.00	4.00	4.00
*A. fumigatus*		33.33	33.33	4.00
*C. neoformans*		7.69	33.33	7.69

Antimicrobial activity exhibited by naturally occurring flavonoids is attributed to the presence of a phenolic group, and the addition of more such groups might potentiate the activity [28]. However increasing the number of hydroxyl, methoxyl or glycosyl substituents leads to a steady loss of antifungal activity [29]. This observation may possibly explain the low activity exhibited by PFA in this study. Other reports [30] suggest that the organism (*Verticillium albo-atrum*) used by [29] may be exceptional in its response to hydroxyl/methoxyl substitution. Although the fungal pathogens used in that study were plant pathogens, it is not clear whether the response of the pathogens used in this study were influenced by such substitutions. It may be likely that the structure-activity relationship of antifungal compounds could possibly be associated with multiple factors, unlike in bacteria where cell wall interactions are most critical. Variation in time interval and susceptibility of *C. albicans* to TMA was observed. This difference in time of incubation suggests that *C. albicans* might have overcome the antifungal effect of TMA and was only susceptible at higher concentrations. This observation may suggest a possible fungistatic effect of TMA on *C. albicans* after 24 h of incubation*.* It is also noteworthy that the very slight change in MIC with prolonged time of incubation suggests that the antifungal effect of the compound is long lived.

### Toxicity studies of compounds

The mutagenic properties of organic substances, whether synthetic or natural, can be tested using the Ames test [31]. The Ames test is based on a short-term bacterial reverse mutation assay aimed at detecting ranges of chemical substances capable of producing genetic damage with resultant gene mutations. The results from the Ames test performed on the isolated compounds are presented in Table 5 as the mean number of revertants per plate in *S. typhimurium* strains TA98 and TA100 ± S.E.M. TMA and IGG were tested for their potential genotoxic effects in independent repeated assays. PFA was not tested in the Ames test due to the limited quantity isolated. Substances are considered active if the number of induced revertant colonies is twice the number of revertant colonies of the negative control (blank) [24]. None of the compounds investigated were mutagenic in the *Salmonella*/microsome tester strains TA98 and TA100. Flavonoid-induced mutation in the Ames test is reported to more or less match that of structurally related compounds, e.g. naphthalene derivatives, and the pathological consequences of mutation occurring from the eating of flavonoid-containing foods is said to be low [32, 33]. The observations are consistent with findings in this study where compounds tested exhibited no mutagenic effect (Table 5).

**Table 5:** **Number of his + revertants in** ***Salmonella typhimurium*** **strains TA98 and TA100 produced by isolated compounds**

		TA98			TA98	
	No. of colonies			No. of colonies		
	Concentration (μg/ml)			Concentration (μg/ml)		
Compounds	1000	100	10	1000	100	10
TMA	23 ± 5.3	28.3 ± 3.2	26.7 ± 3.8	176 ± 31.8	139 ± 2.5	138 ± 67
IGG	25.3 ± 4.6	25.3 ± 4.6	25.5 ± 2.1	170.3 ± 225	169 ± 14.6	154 ± 4.4
PFA	N/D	N/D	N/D	N/D	N/D	N/D
Spontaneous	19.3 ± 4			152 ± 10		
4NQO	170.3 ± 20			960 ± 35.1		

N/D = Not determined.

A similar non-toxic effect was also observed in the cytotoxicity assay when CRFK, Vero and bovine dermis cells were exposed to the test compounds indicating no differences between the three cell lines with regard to their sensitivity to the compounds (Table 6). Although the influence of structural-activity relationships on cytotoxicity is not well understood [34] methoxy and hydroxyl groups in biflavonoids and monoflavonoids, may play a crucial role in mediating cytotoxic activity [34]. This may possibly explain the observed non-toxic effect of the test compounds.

**Table 6:** **Cytotoxicity of isolated compounds on different cell types**

	Compounds (μg/ml)		
Cells	TMA	IGG	PFA
Vero	>1000	>1000	N/D
CRFK	>1000	>1000	N/D
Bovine dermis	>1000	>1000	N/D

N/D = Not determined.

## Conclusion

Compound IGG was the most active against *E. coli*, *S. aureus, A. fumigatus* and *C. neoformans*, exhibiting both antibacterial and antifungal activity with good selectivity index values. Compound PFA presented a broad spectrum of activity against *E. faecalis* and *P. aeruginosa*. It could therefore be ascertained that the relationship between structures of the compounds and observed biological activity and toxic effect could support the relevance of functional group substitution in the biological activity of biflavonoids. TMA and IGG had no deleterious effect in the cytotoxicity assay on various cell lines, and mutagenicity studies indicated the putative non-genotoxic effect of these compounds. Further studies, including those incorporating a metabolic activation step, are necessary to confirm this conclusion. Naturally occurring pure compounds exhibiting good antimicrobial activity which can selectively kill microorganisms without being significantly toxic to host cells can be a useful tool in evaluating the potential toxic effect of compounds *in vivo*.

The relatively high selectivity index indicates that these compounds or the extracts from which they were isolated may be useful in managing bacterial and fungal infections in animals and humans.

## Abbreviations

Amp-B: Amphotericin B
CC_50_: Cytotoxicity (50% killing effect)
DMSO: Dimethyl sulphoxide
SI: Selectivity index
CRFK: Crandell feline kidney cells
IGG: Isoginkgetin
INT: p-iodonitrotetrazolium violet
4-NQO: Nitroquinoline-1-oxide
PBS: Phosphate buffered saline
SD: Sabouraud dextrose
TMA: 7, 4’, 7”, 4”’-tetramethoxy amentoflavone
MH: Müller-Hinton
MIC: Minimum inhibitory concentration
MTT: 3-[4,5-dimethylthiazol-2-yl]-2,5-diphenyltetrazolium bromide
N/D: Not determined
PFA: podocarpus flavone–A.
